# Revisiting the hydrogenation behavior of NdGa and its hydride phases

**DOI:** 10.1107/S1600576724000554

**Published:** 2024-02-16

**Authors:** Vitalii Shtender, Johan Cedervall, Gustav Ek, Claudia Zlotea, Mikael S. Andersson, Pascal Manuel, Martin Sahlberg, Ulrich Häussermann

**Affiliations:** a Department of Chemistry – Ångström Laboratory, Uppsala University, Box 538, Uppsala 751 21, Sweden; b Université Paris-Est Creteil, CNRS, ICMPE, UMR 7182, 2 rue Henri Dunant, Thiais 94320, France; c ISIS Neutron and Muon Facility, Science and Technology Facilities Council, Rutherford Appleton Laboratory, Oxford OX11 0QX, United Kingdom; d Department of Materials and Environmental Chemistry, Stockholm University, Stockholm 106 91, Sweden; Ecole National Supérieure des Mines, Saint-Etienne, France

**Keywords:** intermetallic compounds, metal hydrides, crystal structure, Zintl phases

## Abstract

NdGa hydride and deuteride phases were prepared from high-quality NdGa samples and their structures characterized by powder and single-crystal X-ray diffraction and neutron powder diffraction. NdGa with the orthorhombic CrB-type structure absorbs hydrogen at hydrogen pressures ≤ 1 bar until reaching the composition NdGaH(D)_1.1_, which maintains a CrB-type structure. At elevated hydrogen pressure additional hydrogen is absorbed and the maximum composition recovered under standard temperature and pressure conditions is NdGaH(D)_1.6_ with the *Cmcm* LaGaH_1.66_-type structure. This structure is a threefold superstructure with respect to the CrB-type structure. The hydrogen atoms are ordered and distributed on three fully occupied Wyckoff positions corresponding to tetrahedral (4*c*, 8*g*) and trigonal–bipyramidal (8*g*) voids in the parent structure. The threefold superstructure is maintained in the H-deficient phases NaGaH(D)_*x*_ until 1.6 ≥ *x* ≥ 1.2. At lower H concentrations, coinciding with the composition of the hydride obtained from hydrogenation at atmospheric pressure, the unit cell of the CrB-type structure is resumed. This phase can also display H deficiency, NdGaH(D)_*y*_ (1.1 ≥ *y* ≥ 0.9), with H(D) exclusively situated in partially empty tetrahedral voids. The phase boundary between the threefold superstructure (LaGaH_1.66_ type) and the onefold structure (NdGaH_1.1_ type) is estimated on the basis of phase–composition isotherms and neutron powder diffraction to be *x* = 1.15.

## Abstract 2

Detailed investigation of the NdGa–H(D)_2_ systems led to the unambiguous identification of two distinct phases. It was found that NdGaH(D)_*y*_ (1.1 ≥ *y* ≥ 0.9) has a onefold structure while NdGaH(D)_*x*_ (1.6 ≥ *x* ≥ 1.2) adopts a threefold superstructure.

## 1. Introduction

Zintl phases are compounds which constitute an active metal [alkali, alkaline earth (AE) or rare earth (RE)] and a more electronegative *p*-element component (Haussermann *et al.*, 2011 ▸). Like less-polar intermetallic compounds, Zintl phases can react with hydrogen to form hydrides. Although the H content of Zintl phase hydrides is comparatively low (H/*M* < 1), the chemical structures and physical properties of Zintl phases can change profoundly upon H incorporation. The hydrogenation-induced metal–semiconductor transition for the systems AETt–AETtH (AE = Ba and Sr, Tt = Si, Ge, Sn and Pb) (Auer *et al.*, 2017 ▸) and AETrTt–AETrTtH (Tr = Al, Ga, In; Tt = Si, Ge, Sn) (Häussermann, 2008 ▸) has been well investigated. More recently, interest has shifted to RE metal-containing Zintl phases because of hydrogenation-induced magnetic property changes (Werwein *et al.*, 2018 ▸, 2019 ▸; Nedumkandathil *et al.*, 2016 ▸). For example, NdGa, GdGa and Eu_3_Si_4_ are all ferromagnetic Zintl phases which, upon H incorporation, either become antiferromagnetic [NdGa (Ångström *et al.*, 2016 ▸) and GdGa (Nedumkandathil *et al.*, 2016 ▸)] or paramagnetic (Eu_3_Si_4_) hydrides (Ek *et al.*, 2019 ▸). Our main interest resides in the explicit elucidation of the magnetic structures of NdGa and its hydrides, *i.e.* hydrogen-induced magnetic property changes. However, during attempts to prepare pure samples for neutron diffraction studies of the magnetic structure we encountered some peculiarities and deviations from previous work. Consequently, in this paper we want to focus on the structural analysis and provide an updated and clear view of the hydrogenation behavior and hydride phases of NdGa.

The structures of NdGa and its hydrides have previously been studied by Dwight *et al.* (1967 ▸) who concluded that NdGa crystallizes in an orthorhombic CrB-type structure. In the first report on hydrides (and deuterides) of NdGa by Ångström *et al.* (2016 ▸), the hydride NdGaH_∼1.66_ was produced after hydrogenation at 10–20 bar and near 573 K and a monoclinic structure was deduced from neutron powder diffraction (NPD) of its corresponding deuteride. In the NdGaH_1.66_ structure the arrangement of the metal atoms of the NdGa Zintl-phase precursor is essentially maintained while H is incorporated into two distinct positions: a fully occupied tetrahedral void defined by four Nd atoms and a partially occupied (66%) trigonal–bipyramidal void defined by three Nd and two Ga atoms. It was further shown that H de­sorption from NdGaH_1.66_ occurs in two discrete steps that correlate with the two types of H position. Later, Auer *et al.* (2019 ▸) studied the formation of NdGaD*_x_* by *in situ* NPD and concluded that the structure of NdGaH_1.66_ is an orthorhombic threefold superstructure of the CrB-type structure of the parent compound NdGa, with H atoms ordered on three fully occupied positions. In addition, Auer *et al.* (2019 ▸) found evidence of twofold superstructuring for lower H concentrations, 1.53 < *x* < 1.66, whereas phases with H compositions 1.10 < *x* < 1.22 attained the cell of the CrB-type structure and hydrides with H contents lower than NdGaH_*x*_ (*x* < 1) were not reported.

In the previous reports on NdGa hydrides the authors have experienced difficulties with preparing impurity-free NdGa samples (Auer *et al.*, 2019 ▸; Ångström *et al.*, 2016 ▸). The presence of impurities can impede the structure characterization of hydrides from powder diffraction and may also lead to misinterpretation of magnetic properties from magnetometry measurements. In this article we report on the synthesis of (virtually) phase-pure and single-crystalline NdGa and NdGaH(D)_*x*_ hydrides/deuterides (*x* = 0.9, 1.1, 1.2 and 1.6). The single-crystal nature and the phase purity of the samples allowed for more accurate structure refinements and a more reliable analysis of the superstructure phenomenon.

## 2. Experiment

### 2.1. Synthesis

NdGa was synthesized by arc melting pieces of pure Nd (Smart-elements, purity 99.9%) and Ga (99.999%) in an Ar atmosphere (99.999%) with several remelts. A 1% excess of Nd was used to avoid the presence of NdGa_2_ impurities. The sample was wrapped in Ta foil, sealed in a stainless steel tube under Ar, annealed at 1173 K for 5 d and quenched in cold water. The sample was then crushed, and small single crystals were manually collected from this powder. The powder and the single crystals were used for further characterization.

Hydrides and deuterides were synthesized similarly to previous work (Auer *et al.*, 2019 ▸; Ångström *et al.*, 2016 ▸). For the hydrogenation, coarse pieces of the sample were loaded into a stainless steel autoclave. Prior to the actual hydrogenation an activation procedure at 573 K in a vacuum was performed. The hydrogenation was started at 573 K and small portions of H_2_ (steps of 0.2–0.5 bar) were introduced into the autoclave. This procedure is needed to avoid hydrogen-induced disproportionation of NdGa, since the kinetics of absorption are fast at the beginning. This procedure also prevents the formation of any NdH*_x_* as observed for other RE-rich compounds (Shtender *et al.*, 2015 ▸). After the active absorption at 573 K had finished, the temperature was lowered to room temperature and the H_2_ pressure was increased to 25 bar. The absorption process was considered complete when no obvious pressure changes could be observed after waiting for a couple of hours. Pressure–composition isotherms (PCIs) were measured in an in-house-built Sieverts-type instrument with thermostatically calibrated volumes at 298 K. Several coarse pieces of the sample were placed inside a stainless steel sample holder and activated by a heat treatment at 573 K under a dynamic vacuum for 6 h. The temperature was maintained at 573 K, and small doses of gaseous hydrogen were introduced to avoid hydrogen-induced disproportionation, as explained above. For the calculation of the hydrogen capacity, the real equation of state for hydrogen was used from the program *GASPAK* (Version 3.32; Cryodata Inc., Boulder, Colorado, USA). This apparatus was also used to prepare NdGaH_*x*_ samples with *x* = 1.1, 1.2 and 1.6 at 1 bar and room temperature. The NdGaH(D)_*x*_ single crystals were collected from the hydrogenated powder in a similar manner to the parent compound.

### 2.2. Thermal de­sorption experiments

The thermal de­sorption of NdGaH_1.6_ and NdGaD_1.6_ was studied by simultaneous differential scanning calorimetry (DSC). A small quantity (10–20 mg) of powder was placed into an Al_2_O_3_ crucible which was purged three times with Ar (99.999%). The de­sorption experiments were performed by heating the sample to 873 K in an Ar atmosphere at a heating rate of 20 K min^−1^. The same protocol was used to estimate the formation temperature for the NdGa compound.

Thermal de­sorption spectrometry (TDS) experiments were conducted on fully saturated hydrides and deuterides (up to 873 K at a heating rate of 10 K min^−1^) to measure the hydrogen/deuterium de­sorption profiles with temperature. Samples with intermediate hydrogen concentrations were prepared by partial de­sorption using TDS and DSC instruments (by heating to specific temperatures, *e.g.* 473, 523, 548, 573 and 648 K) for *ex situ* single-crystal X-ray diffraction. NdGaD_0.9_ deuteride for NPD diffraction was prepared by de­sorption of a fully saturated sample with the TDS technique by heating up to 648 K.

Thermal de­sorption from the fully saturated hydride was also studied with *in situ* synchrotron radiation powder X-ray diffraction (SRPXRD) on the P02.1 beamline at PETRA III at the German Electron Synchrotron (DESY, Hamburg, Germany) using a Varex XRD 4343CT detector, a sample-to-detector distance of 1150 mm and a fixed wavelength of 0.20734 Å as determined by an LaB_6_ standard. The powder was loaded into a quartz tube mounted on a gas cell as described previously (Höglin *et al.*, 2015 ▸; Jensen *et al.*, 2010 ▸) and heated under vacuum at 10 K min^−1^ using a filament wrapped around the quartz tube. In addition, SRPXRD patterns for NdGa, NdGaD_0.9_, NdGaH_1.1_, NdGaH_1.2_ and NdGaH_1.6_ were collected at room temperature (this was done to eliminate the strong preferred orientation present for the in-house diffractometer). The data were integrated using the software *Dawn* (Filik *et al.*, 2017 ▸). Unit-cell parameters were extracted by sequential Pawley refinements in the software *TOPAS* (Version 6; Coelho, 2018 ▸) with the background described by a fifth-order Chebyschev polynomial and peak shapes by Thompson–Cox–Hastings pseudo-Voigt functions.

### 2.3. Phase analysis

The phase purity of the NdGa samples was checked with powder X-ray diffraction (PXRD) and a scanning electron microscope equipped with an energy-dispersive X-ray spectroscopy (EDS) detector. The powders were mounted on single-crystal Si sample holders and X-ray diffraction patterns were collected using a Bruker D8 Advance with Cu *K*α radiation at room temperature. The Rietveld method (Rietveld, 1969 ▸) as implemented in *TOPAS* (Version 6; Coelho, 2018 ▸) was used for analysis of the diffraction data. The samples for electron microscopy analysis were prepared by standard metallographic techniques by grinding with SiC paper. For qualitative determination of the impurity, X-ray photoelectron spectroscopy (XPS) data were collected using a PHI Quantera II system with an Al *K*α X-ray source and a hemispherical electron energy analyzer having a pass energy of 26.00 eV. Before recording an XPS spectrum the sample surface was cleaned by 500 eV Ar-ion sputtering for 30 s.

For all samples, single crystals were analyzed. A Bruker D8 single-crystal X-ray diffractometer with Mo *K*α radiation (λ = 0.71073 Å) upgraded with an Incoatec Microfocus Source (IμS) and an APEXII CCD area detector was utilized to collect single-crystal X-ray diffraction (SCXRD) patterns at room temperature. The SCXRD data reduction and numerical absorption corrections were performed using *SADABS* (Krause *et al.*, 2015 ▸) as implemented in the *APEX3* software (Bruker, 2016 ▸). Initial models of the crystal structures were first obtained with the program *SHELXT2019* (Sheldrick, 2015*a* ▸) and refined using the program *SHELXL2019* (Sheldrick, 2015*b* ▸) within the *APEX3* software package.

Neutron powder diffraction (NPD) patterns of the deuterides (NdGaD_0.9_ and NdGaD_1.6_) were collected using the instrument WISH at the ISIS Neutron and Muon Source (Didcot, UK) (Chapon *et al.*, 2011 ▸). Around 3 g of each sample were placed in separate V cans and measured at 20 K. The obtained diffraction data were analyzed using the Rietveld method (Rietveld, 1969 ▸) implemented in the software *FULLPROF* (Rodriguez-Carvajal *et al.*, 1999 ▸). The deuterium positions were determined using difference Fourier maps. In the refinements several structure parameters, including the atomic positions, atomic displacement and D occupancies, were allowed to vary.

## 3. Results and discussion

### 3.1. Synthesis of (nearly) phase-pure NdGa and its hydrogenation/de­sorption behavior

**Figure 1:**
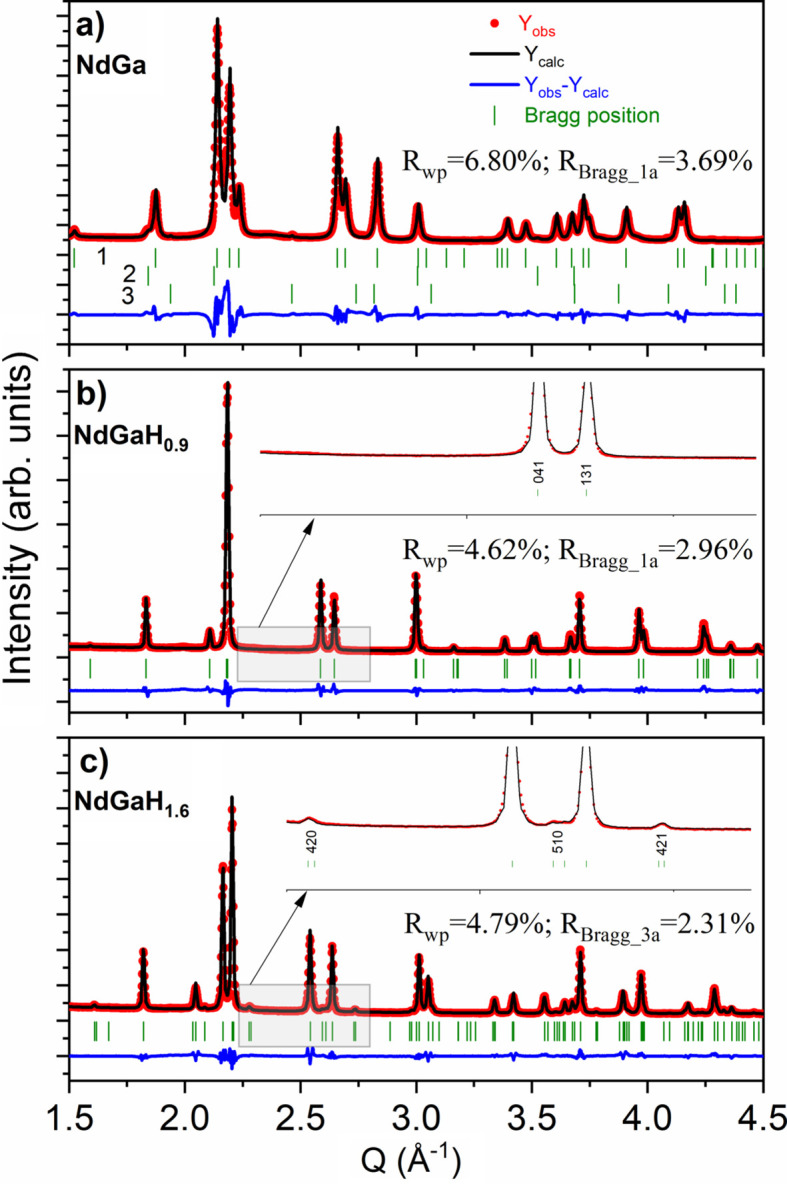
Rietveld refinement for the SRPXRD data profiles for NdGaH_*x*_ (*x* = 0, 0.9 and 1.6) compounds. The insets show the absence and presence of the 3*a* superlattice peaks for *x* = 0.9 and *x* = 1.6, respectively. In panel (*a*), 1 stands for 98% NdGa, 2 is 1.5% Nd_1−*x*_Ce_*x*_O_1.75_ and 3 is 0.5% TaGaO_4_.

Some available phase diagrams of the Nd–Ga system (Villars *et al.*, 2018 ▸) report that the binary NdGa compound forms from the peritectic reaction of NdGa_2_ and melt at around 1173 K (Okamoto, 1990 ▸; Manory *et al.*, 1978 ▸). Thus, the synthesis of NdGa is typically performed by arc melting stoichiometric amounts of elemental metals and subsequent long annealing at temperatures below ∼1173 K, and yet the obtained NdGa products are notoriously accompanied by (unknown) impurities (Auer *et al.*, 2019 ▸; Ångström *et al.*, 2016 ▸). However, in the phase diagram proposed by Yatsenko *et al.* (1979 ▸) for the Nd–Ga system, the peritectic line is at a much higher temperature of 1361 K, which is in good agreement with the temperature found from our present DSC experiments (see Fig. S1 in the supporting information). Thus, higher annealing temperatures can be applied. It was found that an increase in the annealing temperature to 1173 K effectively removes the unknown impurities. It was also found that the purity of the Nd metal plays a very important role in the synthesis of high-quality NdGa samples (it should not be lower than 99.9%) and Nd should be strictly handled in an Ar-filled glove box to avoid oxidation. As a result, the NdGa alloy was synthesized with a much higher purity than in the previous reports, although the samples were still contaminated with up to ∼1.5 wt% of Nd_1−*x*_Ce*_x_* O_1.75_ (Ce is a common impurity in Nd and its presence was found by EDS). Another impurity was observed from XPS which detected peaks related to Ta (for EDS the Ta peaks are hidden in the background). Rietveld refinements of the PXRD data for NdGa, presented in Fig. 1 ▸(*a*), revealed the presence of ∼0.5 wt% TaGaO_4_ (most probably due to the synthesis in Ta foil). Fig. 1 ▸ also presents Rietveld refinements of PXRD data for the two hydrides NdGaH_0.9_ and NdGaH_1.6_. As shown in Fig. S2, our NdGa sample consisted of crystals with sizes ranging from 20 to 150 µm, and EDS analysis of the crystals gives an average composition of Nd_1.02 (1)_Ga_0.98 (1)_, confirming that stoichiometric NdGa was synthesized.

**Figure 2:**
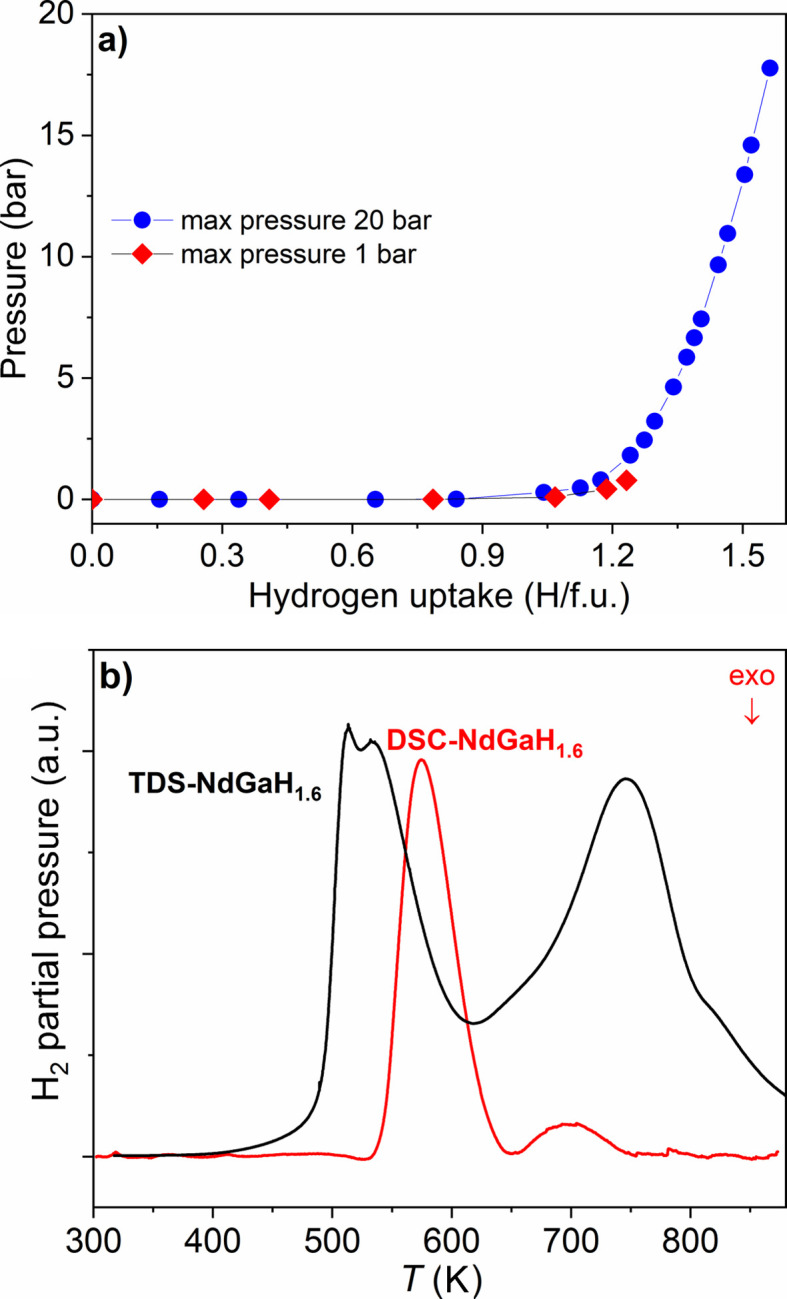
(*a*) PCI curves for NdGa at 573 K. (*b*) Combined TDS and DSC curves for NdGaH_1.6_.

PCIs recorded at 573 K with H_2_ [Fig. 2 ▸(*a*)] confirmed the results from previous work: NdGa behaves as a hydrogen getter (*i.e.* it absorbs H without overpressure) until a composition of NdGaH_1.1_ is reached at around 1 bar. At 20 bar a hydrogen capacity of 1.56 H per formula unit (f.u.) is observed. Using the same conditions, Ångström *et al.* (2016 ▸) reported a limiting composition of NdGaH_2_ after 48 h. Auer *et al.* (2019 ▸) obtained 1.8 D f.u.^−1^ at 40 bar and 473 K in their *in situ* NPD study. However, compositions with >1.65 H f.u.^−1^ can only be observed *in situ* (*i.e.* at H_2_ pressure). The maximum H content in recovered NdGa hydride (under standard temperature and pressure conditions) has been consistently reported as ∼1.6 H f.u.^−1^ and henceforth this phase is termed NdGaH(D)_1.6_. The TDS and DSC results presented in Fig. 2 ▸(*b*) suggest a ‘two-step’ de­sorption process for NdGaH_1.6_ with maximum de­sorption rates at ∼523 and ∼723 K as proposed by Ångström *et al.* (2016 ▸). The temperature discrepancies between the two methods can be attributed to the fact that the TDS experiments were performed in a vacuum while the DSC experiments were done using an Ar atmosphere for which partial oxidation at higher temperatures might occur. The doublet feature of the first TDS peak is seen for both the hydride and the deuteride sample and appears to be independent of the heating rate (Ångström *et al.*, 2016 ▸). This phenomenon will be addressed in more detail in the next section. The large temperature separation between the two de­sorption steps suggests that it should be possible to prepare the distinct monohydride phase NdGaH.

Interestingly, the single-crystalline nature of the Zintl-phase starting material is preserved after both hydrogenation and de­sorption for both the hydrides and the deuterides (Fig. S2), allowing conclusive SCXRD studies of both fully hydrogenated and partially desorbed samples. As previously described, the NPD study by Auer *et al.* (2019 ▸) found evidence of a threefold superstructure for NaGaD_1.6_ at 480 K and 98 bar D_2_ pressure, which corresponds to the LaGaH_1.66_ structure. From the PXRD and SCXRD patterns presented in Figs. 1 ▸(*c*) and 3 ▸ for NdGaH_1.6_, it can be concluded that this superstructure is maintained under ambient conditions. Thus, the monoclinic structure originally put forward for NdGaH(D)_1.6_ by Ångström *et al.* (2016 ▸) can be ruled out.

In order to examine whether there are distinct intermediate hydride phases, as suggested by Auer *et al.* (2019 ▸), several controlled de­sorption experiments were performed using both DSC (Ar atmosphere, for hydrides) and TDS (vacuum, for deuterides) at various temperatures. The selection of temperatures for these experiments was based on the TDS peak evolution presented in Fig. 2 ▸(*b*). Desorption experiments up to 648 K in a dynamic vacuum, as described in the *Experiment* section[Sec sec2], aimed at complete removal of the H atoms within the trigonal–bipyramidal Nd_3_Ga_2_ environment to produce the distinct stoichiometric phase NdGaH(D) [which was not achieved in the de­sorption experiment at 493 K by Auer *et al.* (2019 ▸)]. However, the hydrogen compositions of products obtained by partial de­sorption are uncertain and further characterization is needed to determine the hydrogen (deuterium) content. The NPD experiment on the sample NdGaD_*x*_ prepared by de­sorption of NdGaD_1.6_ to 648 K revealed that the true composition is NdGaD_0.9_ and, on the basis of this result, it was assumed that the hydrogenated sample synthesized under identical conditions has the same composition. A more detailed analysis of the NPD results is presented in Section 3.3. To produce accurate compositions in the concentration range 1 < *x* < 1.6, NdGaH_1.1_ and NdGaH_1.2_ were synthesized in a Sievert-type apparatus acquiring PCI curves at low equilibrium pressures.

### 3.2. Crystal structure variations as a function of H content

**Figure 4:**
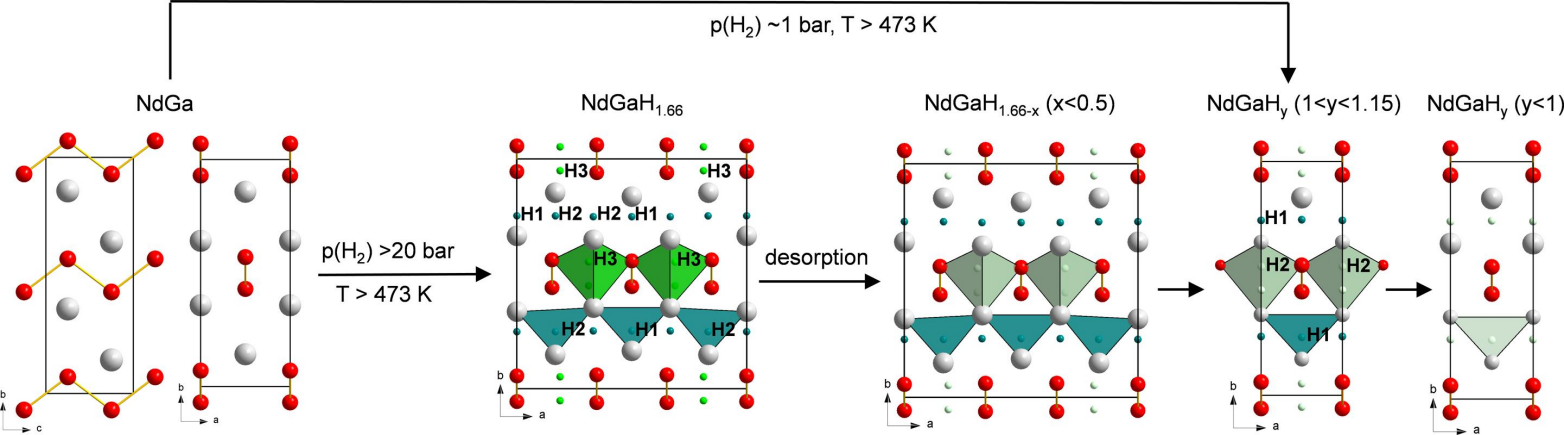
Crystal structures of (left) NdGa and (middle to right) its hydride phases NdGaH_1.6−*x*_ (*x* < 0.5), NdGaH_*y*_ (1 < *y* < 1.15) and NdGaH_*y*_ (*y* < 1), respectively. Nd and Ga atoms are depicted as gray and red circles, respectively. H atoms and their metal coordination polyhedra are represented in green (trigonal–bipyramidal interstitial) and teal (tetrahedral interstitial). Light green indicates partial occupancy. Formation conditions for the hydride phases are indicated as arrows.

Fig. 4 ▸ shows the crystal structures and summarizes the results from SCXRD experiments for NdGa, NdGaH_0.9_, NdGaH_1.1_, NdGaH_1.2_ and NdGaH_1.6_ (for which the H concentrations can be considered more or less reliable as discussed above). Details for the SCXRD-determined structures are presented in Tables 1 ▸ and 2 ▸.

The crystal structure of the Zintl-phase NdGa features polyanionic zigzag chains of Ga atoms, which are formally in a reduced Ga^3−^ state and thus singly bonded, while carrying two lone electron pairs that are hosted in the *p_x_* and *sp* ^2^ orbitals. The chains run along the crystallographic *c* direction and are embedded in slabs of trigonal prisms formed by the Nd atoms which run along the *a* direction (Hyde & Andersson, 1989 ▸). The two types of voids for H, tetrahedral H@Nd_4_ and trigonal–bipyramidal H@Nd_3_Ga_2_, are 4*c* (0, ∼¾, ¼) and 4*c* (0, ∼0.55, ¼), respectively.

**Figure 5:**
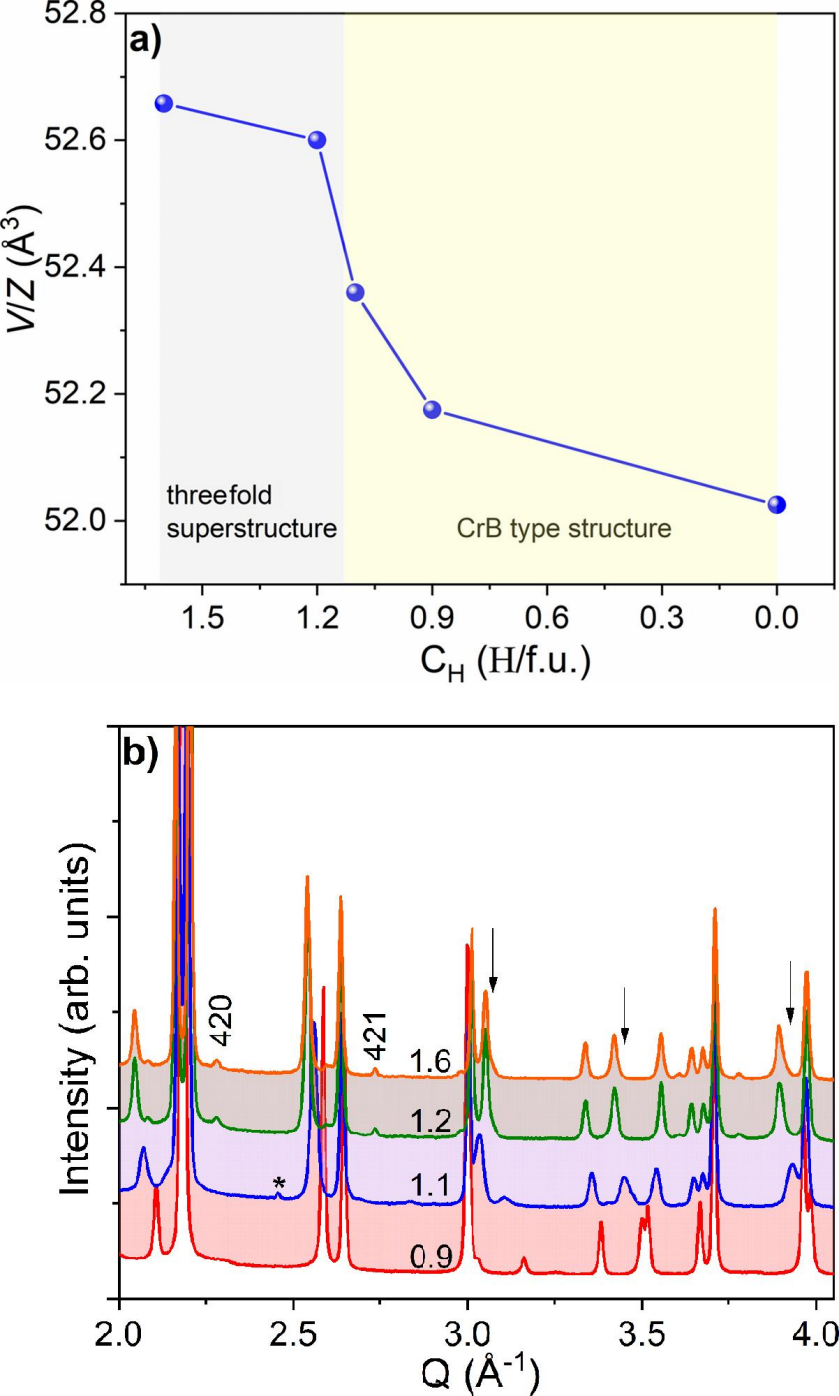
(*a*) *V*/*Z* versus H f.u.^−1^ for hydrides with known H content. (*b*) A comparison of patterns, with broader peaks for NdGaH_1.1_. The labels 420 and 421 are superstructure reflections, arrows show broadened peaks, and the asterisk (*) indicates the impurity of TaGaO_4_.

Hydrogenation of NdGa at pressures above 20 bar results in the formation of NdGaH_1.6_, where the *a* axis of the initial CrB-type structure is tripled (*a* = 3*a*_CrB_) while the *Cmcm* space group type symmetry is maintained. The tetrahedral voids of the CrB-type structure are completely filled (positions H1 and H2 in Fig. 4 ▸) and two-thirds of the trigonal–bipyramidal voids are filled in an orderly fashion (position H3 in Fig. 4 ▸). The *b* lattice parameter of NdGaH_1.6_ is increased by 9% compared with that of NdGa [from 11.246 (3) to 12.264 (2) Å], whereas *a*/3 is decreased by 1.8% (from 4.186 to 4.111 Å) and the *c* parameter (*i.e.* along the direction of the Ga chains) is hardly influenced (∼4.17 Å, *cf.* Table 2 ▸). As a consequence, the volume per formula unit (*V*/*Z*) increases by just 1%, from 52.1 to 52.6 Å^3^. Remarkably, the lattice parameters (and volume) of NdGaH_1.2_ are very similar to those of NdGaH_1.6_, as can be seen in Fig. 5 ▸(*a*), which presents a comparison of the volumes of the different phases (*x* = 0, 0.9, 1.1, 1.2 and 1.6). Below 1.2 H f.u.^−1^ hydrogenated NdGa adopts the CrB-type structure (*i.e.* the 1*a* structure). The evolution of the PXRD patterns from 1.6 to 0.9 H f.u.^−1^ is presented in Fig. 5 ▸(*b*) which, together with Fig. 5 ▸(*a*), provides insights into the boundaries between the different structures. For NdGaH_1.1_ the 3*a* superstructure reflections are absent, while the main reflections appear significantly broadened. Refinement of the PXRD pattern as a two-phase mixture (Fig. S3) gives slightly improved discrepancy values (*R*_wp_), indicating heterogeneity of the NdGaH_1.1_ sample. We note that the NdGaH_1.1_ phase obtained by PCI at low pressure compares well to the NdGaD_1.11_ phase obtained by Auer *et al.* (2019 ▸) from de­sorption of NdGaD_1.6_ at 493 K (1 h, vacuum). The lattice parameters are also comparable: *a* = 4.1656 (3) Å, *b* = 12.0336 (9) Å and *c* = 4.1858 (2) Å for NdGaH_1.1_ versus *a* = 4.1600 (3) Å, *b* = 12.0374 (9) Å and *c* = 4.1825 (3) Å for NdGaD_1.11_. However, the PXRD and SCXRD data show no evidence of the 2*a* superstructure as proposed by Auer *et al.* (2019 ▸). The boundary for the 1*a*–3*a* structural change is assumed to be around 1.15 H f.u.^−1^.

**Figure 6:**
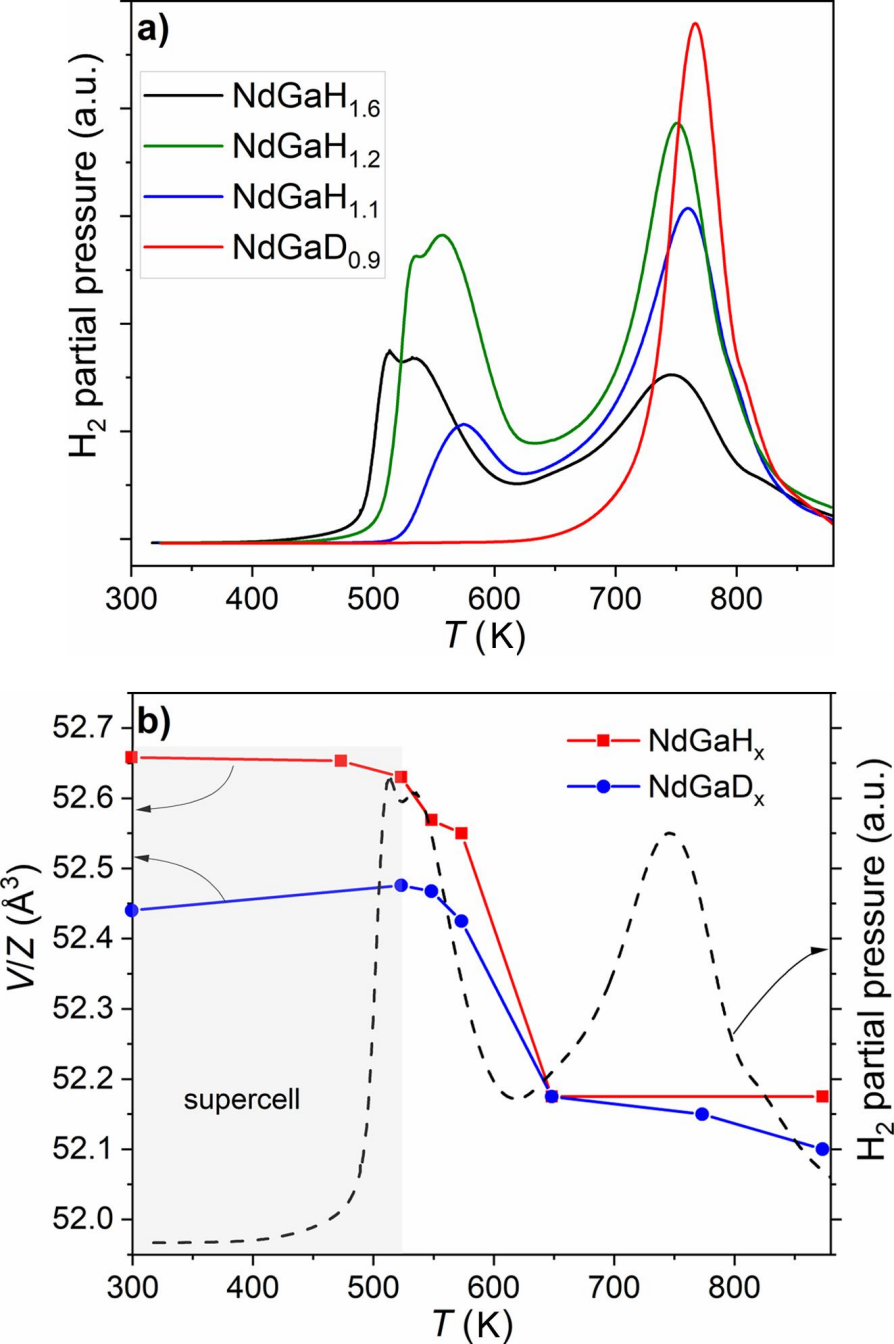
(*a*) TDS spectra and (*b*) *V*/*Z* versus *T* plots showing the region where the supercell exists. NdGaH_1.1_ is a two-phase sample with 1*a* and 3*a* unit cells, while NdGaH_1.6_ is 3*a* phase sample. *T* = 523 K is the last temperature which results in a 3*a* structure, while de­sorption at 573 K results in a 1*a* structure.

Fig. 6 ▸(*a*) summarizes the results from the de­sorption experiments where the thermal de­sorption spectra of NdGaH_1.6_, NdGaH_1.2_, NdGaH_1.1_ and NdGaD_0.9_ are compared. As already mentioned, the first peak in the spectrum of NdGa_1.6_ exhibits a doublet feature which is present for both samples where only the threefold supercell structure of the hydride is present. However, NdGaH_1.1_ does not exhibit any clear doublet feature even though a de­sorption event is still present in this temperature region. The TDS spectrum for NdGaD_0.9_, which has the 1*a* structure, exhibits a single de­sorption step in the same temperature range as the second de­sorption step for NdGaH_1.6_, NdGaH_1.2_ and NdGaH_1.1_. The SCXRD, PXRD and TDS data together suggest that the first peak is associated with de­sorption of H from the trigonal–bipyramidal interstitials, while the second peak is associated with de­sorption from the tetrahedral interstitials. Consequently, the doublet feature mirrors a two-step de­sorption of H from the trigonal–bipyramidal interstitials, corresponding initially to de­sorption from the phase with the 3*a* superstructure until the estimated boundary at around 1.15 H f.u.^−1^ and consecutively from the phase with the 1*a* structure. Additionally, one should note that the onset temperatures of de­sorption for the NdGaH_1.6_, NdGaH_1.2_ and NdGaH_1.1_ samples are different, which implies higher stability of the 1*a* hydride structure.

Fig. 6 ▸(*b*) illustrates the *V*/*Z* values of phases obtained from DSC (hydrides) and TDS (deuterides) de­sorption experiments at various temperatures across the first and second de­sorption peaks. The H(D) contents of these phases are uncertain but the 648 K sample has 0.9 H(D) f.u.^−1^, which was determined using NPD. From PXRD, the temperature boundary for the 3*a*–1*a* structural change was determined to be around 523 K [Figs. 6(*a*) and S4]. This coincides well with the center of the doublet feature in the TDS spectrum. Systematically larger cell volumes were observed for the hydrides than for the corresponding deuterides. However, both hydrides and deuterides exhibit similar changes in their cell parameters with respect to temperature.

**Figure 7:**
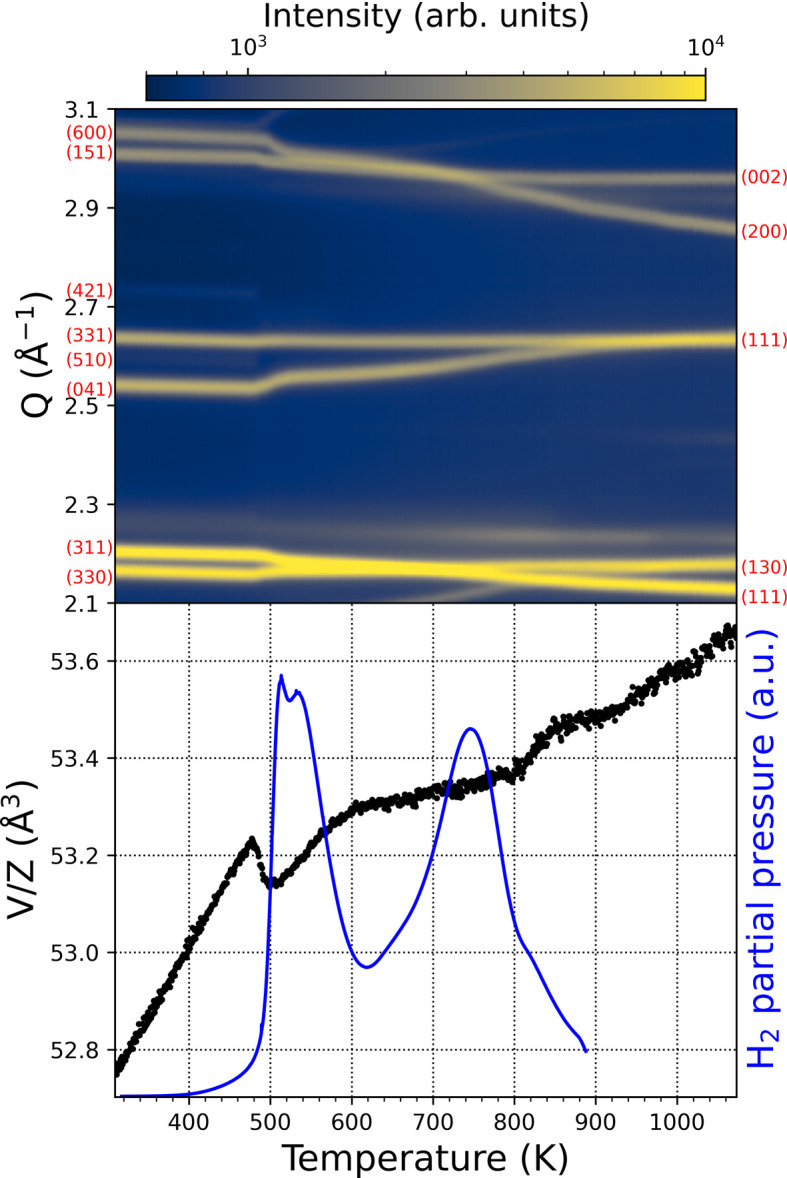
(Top) Selected region of the *in situ* SRPXRD map. The white star indicates the disappearance of the superlattice peak 421. (Bottom) *V*/*Z* with the TDS spectrum for NdGaH_1.6_.

A detailed study of the structural evolution during hydrogen de­sorption was carried out by *in situ* SRPXRD experiments on NdGaH_1.6_ during de­sorption in a vacuum. A selected region of the SRPXRD map together with the *V*/*Z* data and the TDS spectrum for NdGaH_1.6_ are presented in Fig. 7 ▸. From room temperature up to 473 K there is a linear increase in the volume due to thermal expansion. Above 473 K *V*/*Z* drops sharply, indicating the start of de­sorption, which coincides with the onset of the first de­sorption peak in the TDS spectrum (lower panel of Fig. 7 ▸). The superlattice reflection 421 disappears at around 493 K, accompanied by a discontinuous change in the 041 peak. This further indicates that the first peak of the doublet on the TDS curve correlates with the disappearance of the superlattice peaks due to de­sorption. However, the second peak of the doublet is the beginning of de­sorption from the saturated hydride with the 1*a* structure. Furthermore, at this temperature two pairs of main peaks (330 and 311, and 151 and 600) start to overlap, showing further changes in the structure (shrinking of the unit cell due to hydrogen release). Above 493 K only peaks related to the CrB-type structure are present, although they continue to change due to the ongoing de­sorption process. At around 773 K the two peak pairs (330 and 311, and 151 and 600) split and can once again be resolved. At around 873 K the 041 and 331 peaks start to overlap, coinciding with the end of de­sorption. After completion of de­sorption the volume increases linearly with temperature. The TDS and SRPXRD data show good agreement.

To summarize, the X-ray diffraction and de­sorption experiments suggest the existence of the NdGaH(D)_*x*_ (1.6 ≥ *x* ≥ 1.2) phase with the 3*a* superstructure and the NdGaH(D)_*y*_ (1.1 ≥ *y* ≥ 0.9) phase with the 1*a* structure (CrB-type structure). The compositions with *y* = 1.1 and 0.9 correspond to weakly occupied trigonal–bipyramidal interstitials and weakly depleted tetrahedral interstitials, respectively (Fig. 4 ▸). The 1*a* structured phase NdGdH_1.1_ represents both the product of the initial absorption (H getter behavior of NdGa) and the product after the first de­sorption step.

### 3.3. Crystal structures of NdGaD_1.6_ and NdGaD_0.9_ from NPD at 20 K

**Figure 8:**
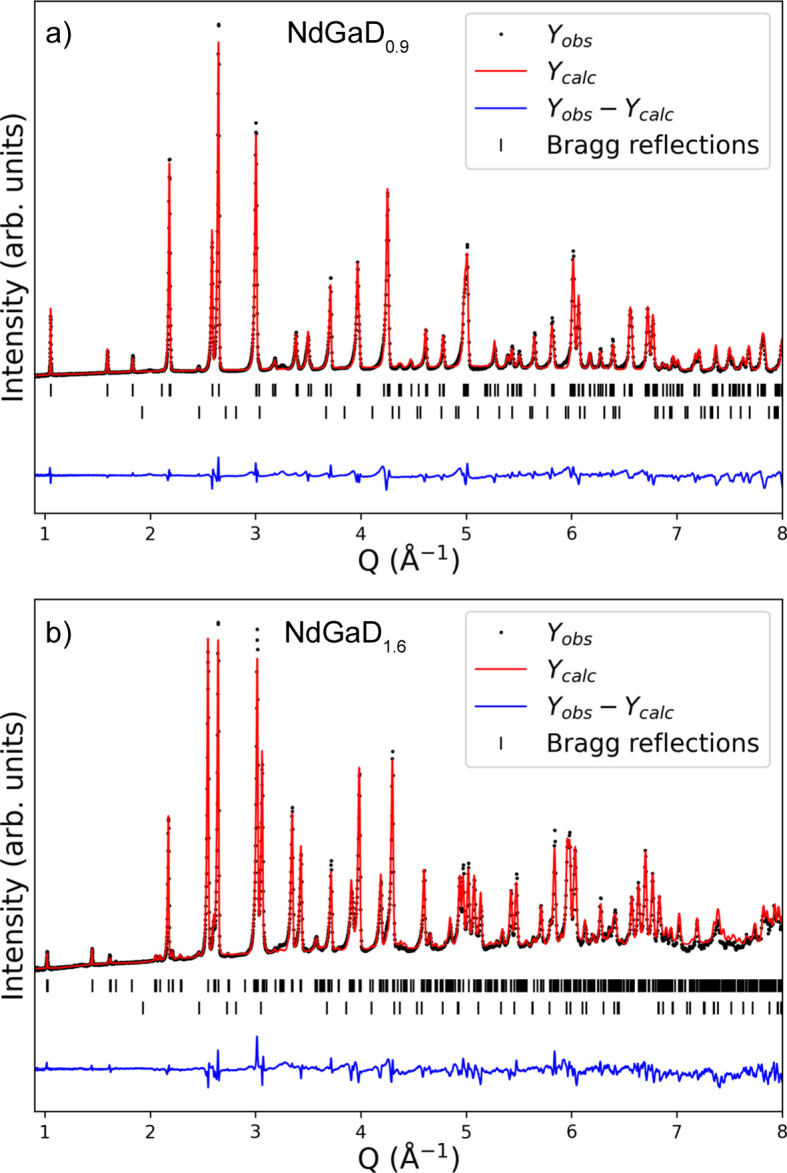
Rietveld refinement of (*a*) NdGaD_0.9_ and (*b*) NdGaD_1.6_ from the 90° detector bank 3 at 20 K with *R*_wp_ = 6.00 and 6.03%, respectively. The bottom rows of Bragg reflections correspond to a secondary phase, TaGaO_4_ (0.5%).

To determine the H(D) positions and occupancies in NdGaH(D)_*x*_ accurately, NPD experiments were performed for two samples with nominal compositions NdGaD and NdGaD_1.6_ at 20 K and ambient pressure. Rietveld refinements of the NPD data revealed that the true compositions of the samples are NdGaD_0.9_ and NdGaD_1.6_. The refinements of the NPD data from detector bank 3 are presented in Fig. 8 ▸ for NdGaD_0.9_ and NdGaD_1.6_, while the structural parameters obtained from the refinements are presented in Table 3 ▸. For NdGaD_1.6_ a 3*a* supercell is found, as previously reported by Auer *et al.* (2019 ▸) for NdGaD_1.66_ at 480 K and 98 bar D_2_ pressure. Furthermore, the observed 3*a* superstructure is in good agreement with the findings from the PXRD and SCXRD results presented in Section 3.1 for NdGaH_1.6_. In the superstructure there are a total of three deuterium positions, where D1 and D2 are tetrahedral interstitials while D3 is a trigonal–bipyramidal interstitial (Fig. 4 ▸). As for the PXRD and SCXRD experiments, no supercell is observed in the NPD experiment for NdGaD_0.9_. This is also in good agreement with the NPD results from Auer *et al.* (2019 ▸), who concluded that there is no supercell for NdGaD_1.11_, NdGaD_1.16_ and NdGaD_1.22_. However, contrary to their results which suggest that NdGaD_1.11_, NdGaD_1.16_ and NdGaD_1.22_ have two deuterium positions, the NPD results presented here show that NdGaD_0.9_ only exhibits a single deuterium position with the D atom as a tetrahedral interstitial (Fig. 4 ▸ and corresponding Fourier difference maps in Fig. S5). The discrepancy between the study by Auer and co-workers and the results presented here could be related to the presence of unidentified impurities in the samples used by (Auer *et al.*, 2019 ▸), together with contamination of the NPD patterns from scattering from the sample environment, which probably limited their NPD analysis. Last but not least, the experimental conditions here (*ex situ* NPD) and in the previous work (*in situ* NPD) are different. Comparing the PXRD, SCXRD and NPD results, it can be concluded that in the studied temperature range neither NdGaD_0.9_ nor NdGaD_1.6_ exhibits any structural phase changes, suggesting that the changes from the 3*a* to the 1*a* cell are only related to the hydrogen (deuterium) content.

As mentioned above, the D atoms occupy the tetrahedral interstitials surrounded by four Nd atoms in the NdGaD_0.9_ phase, while the trigonal–bipyramidal interstitial positions are empty. The D—Nd distances are close to equidistant, 2.368 (1) Å (×2) and 2.382 (1) Å (×2), *i.e.* the tetrahedra remain largely undistorted upon hydrogenation of the tetrahedral interstitials. The Ga—Ga distance in NdGaD_0.9_ is 2.491 (1) Å at 20 K (Table 4 ▸), which is drastically reduced compared with NdGa [2.625 (1) Å at room temperature]. This reduction in the Ga—Ga distance was described earlier and is attributed to the development of π-bonding within the Ga zigzag chain. The introduction of H into the tetrahedral interstitials as H^−^ formally leads to oxidation of the singly bonded zigzag chain formed by the Ga^3−^ species, upon which the antibonding part of the π(*p_x_*–*p_x_*) band is emptied. A phase width of NdGaH_1±*y*_ towards hydrogen-poor compositions has previously been questioned by Auer *et al.* (2019 ▸). However, it appears that harsh de­sorption conditions, especially upon application of temperatures above 623 K, can achieve hydrogen-poor phases such as NdGaH_1−*y*_, although the extent of this phase width is currently unknown.

**Figure 9:**
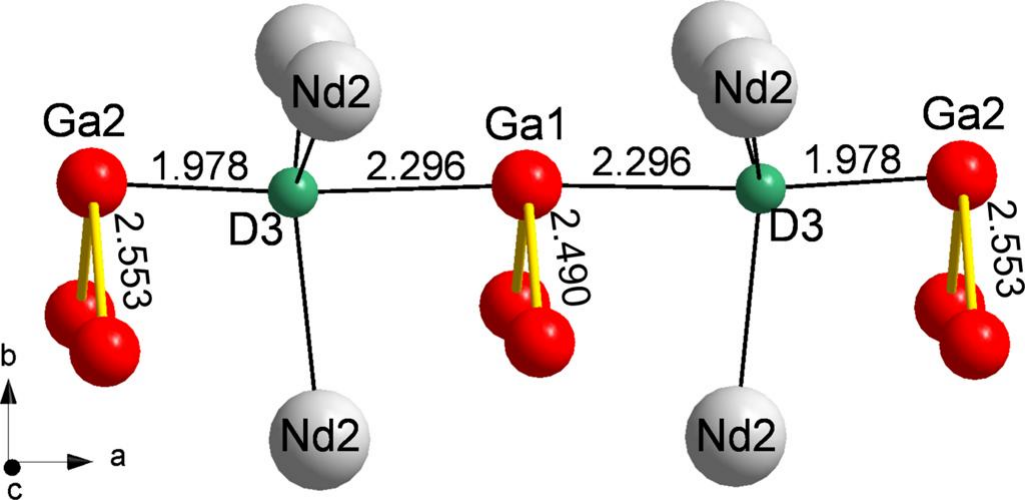
A segment of the NdGaD_1.6_ structure, showing the coordination of D3 atoms in Nd_3_Ga_2_ trigonal–bipyramidal interstitials and the zigzag chains formed by Ga atoms.

The tetrahedral environment of D in NdGaD_1.6_ is more irregular than that of NdGaD_0.9_, with D—Nd distances of 2.327 (1) Å (×2) and 2.396 (1) Å (×2) for D1 [average 2.362 (1) Å] and 2.323 (1) Å, 2.395 (2) Å and 2.420 (1) Å (×2) for D2 [average 2.389 (1) Å]. Most interesting is the irregular trigonal–bipyramidal environment of D3 and the different Ga—Ga distances within the zigzag chains, highlighted in Fig. 9 ▸. The ordered two-thirds occupation of the trigonal–bipyramidal voids in NdGaD_1.6_ (by D3 atoms) implies two crystallographically different zigzag chains, one formed by Ga1 atoms which are coordinated by two D3 atoms (central chain) and one formed by Ga2 atoms which are only coordinated by one D3 atom (peripheral chains). The Ga—Ga distance within the central chains is 2.490 (1) Å (which is similar to that in NdGaD_0.9_), while the Ga—Ga distance within the peripheral chains is substantially elongated [2.553 (1) Å]. The longest Ga—H bonds still being considered single bonds are around 1.7 Å [as in SrGa_2_H_2_ and AEGaTtH; AE = Ca, Sr, Ba; Tt = Si, Ge, Sn (Häussermann, 2008 ▸)], which is substantially shorter than the Ga—D3 distance in NdGaD_1.6_ [Ga2—D3 = 1.978 (1) Å and Ga1—D3 = 2.296 (1) Å]. Yet a distance of 1.978 Å still suggests a covalent Ga—H bonding interaction, albeit weak, which would engage the Ga2 *p_x_* orbital. Thus π(*p_x_*–*p_x_*) bonding within the peripheral zigzag chains is weakened, whereas π-bonding within the central chains remains largely unperturbed (and thus similar to that in NdGaD_0.9_).

There are a few more REGaH(D)_*x*_ phases reported with *Cmcm* 3*a* superstructures: GdGaH_1.66_ (theoretically predicted) (Nedumkandathil *et al.*, 2016 ▸), LaGaD_1.63_ (Werwein *et al.*, 2019 ▸) and TmGaD_0.93_ (Werwein *et al.*, 2019 ▸). For TmGaD_0.93_ (Werwein *et al.*, 2019 ▸), the trigonal–bipyramidal interstitials are not occupied by D atoms, yet a threefold superstructure is realized. Thus, further investigation of REGaH_*x*_ and especially TmGaD_0.9−*x*_ would bring a deeper understanding of the hydrides of Zintl phases as well as their hydrogen-induced property changes.

## 4. Conclusion

The reinvestigation of the hydrogenation behavior of ortho­rhombic NdGa with a *Cmcm* CrB-type structure and the de­sorption behavior of its hydrides NdGaH_*x*_ has led to the unambiguous identification of two distinct phases.

(i) NdGaH(D)_*x*_ (1.6 ≥ *x* ≥ 1.2) adopts the *Cmcm* LaGaH_1.66_-type structure, which is a threefold (3*a*) superstructure with respect to the initial CrB-type structure. The H(D) atoms are located in fully occupied tetrahedral interstitials H@Nd_4_ and flexibly occupy trigonal–bipyramidal interstitials H@Nd_3_Ga_2_ (0.15 < site occupancy factor ≤ 1).

(ii) NdGaH(D)_*y*_ (1.1 ≥ *y* ≥ 0.9) adopts the unit cell of the (1*a*) CrB-type structure of the parent compound NdGa. The hydrogen-rich phase presents weakly occupied trigonal–bipyramidal interstitials and fully occupied tetrahedral interstitials, while the hydrogen-poor phase exhibits no trigonal–bipyramidal interstitials and a weak depletion of the tetrahedral interstitials.

As a result of the multistep de­sorption occurring over a wide temperature range, these phases can be separated by partial de­sorption, making it possible to study in detail the hydrogen- and structure-induced property changes observed for several Zintl phases.

## Supplementary Material

Crystal structure: contains datablock(s) NdGa, NdGaH0.9, NdGaH1.1, NdGaH1.2, NdGaH1.6. DOI: 10.1107/S1600576724000554/nb5371sup1.cif

Structure factors: contains datablock(s) NdGa. DOI: 10.1107/S1600576724000554/nb5371NdGasup2.hkl

Structure factors: contains datablock(s) NdGaH0.9. DOI: 10.1107/S1600576724000554/nb5371NdGaH0.9sup3.hkl

Structure factors: contains datablock(s) NdGaH1.1. DOI: 10.1107/S1600576724000554/nb5371NdGaH1.1sup4.hkl

Structure factors: contains datablock(s) NdGaH1.2. DOI: 10.1107/S1600576724000554/nb5371NdGaH1.2sup5.hkl

Structure factors: contains datablock(s) NdGaH1.6. DOI: 10.1107/S1600576724000554/nb5371NdGaH1.6sup6.hkl

Additional figures. DOI: 10.1107/S1600576724000554/nb5371sup7.pdf

CCDC references: 2265528, 2265529, 2265530, 2265531, 2265532

## Figures and Tables

**Figure 3:**
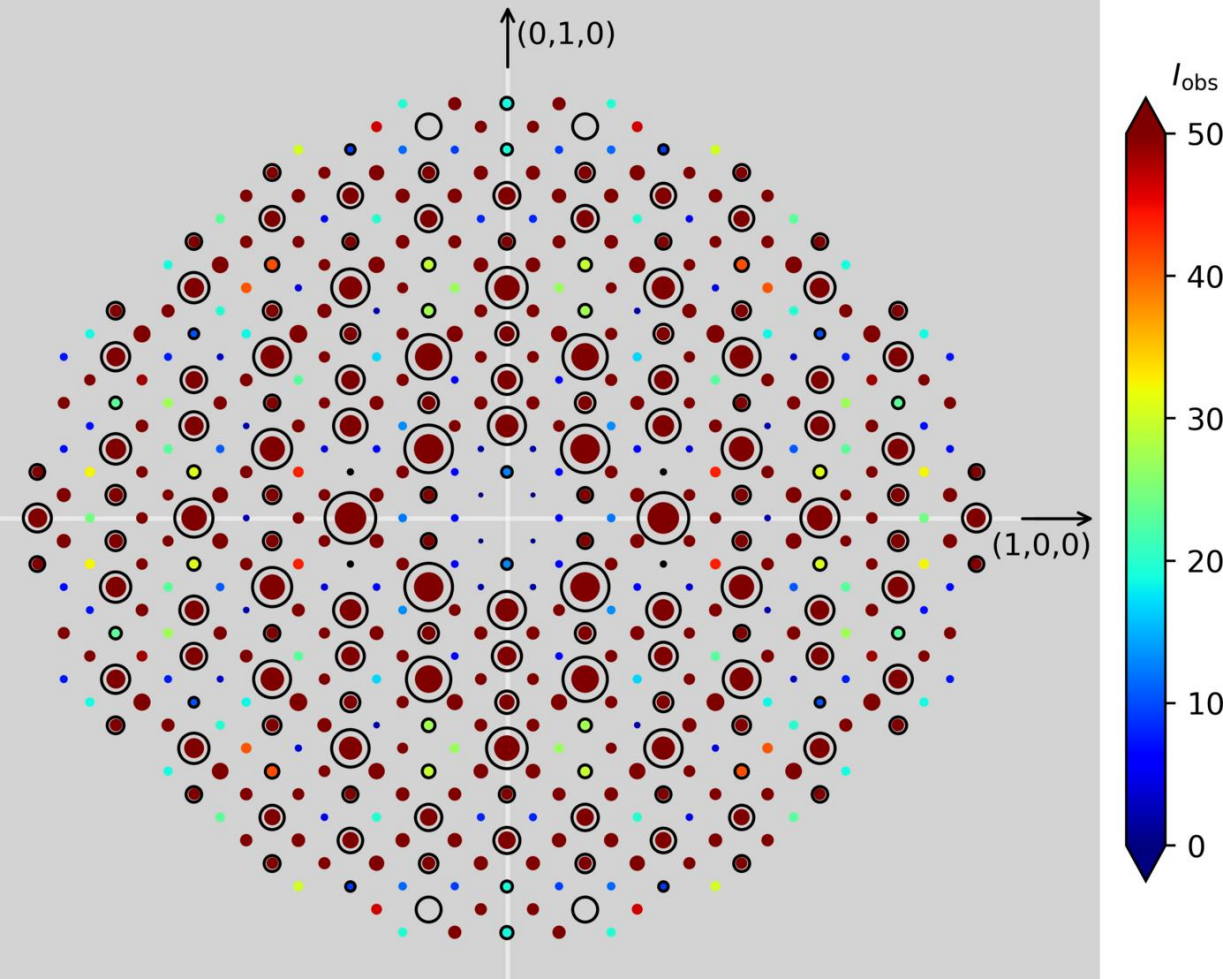
Reconstructed reflections seen for a single crystal of NdGaH_1.6_. The size and color of the dots are correlated with the intensities. The circled reflections represent the CrB-type structure. As can be seen, in between two strong lines (with circles) there are two weaker lines of superstructure reflections related to the threefold structure.

**Table 1:** Crystallographic data and experimental details of the structure determination for NdGaH*_x_* (*x* = 0–1.6) by single-crystal diffraction Experiments were carried out at 296 K with Mo *K*α radiation. All compounds were found to have an orthorhombic structure with space group *Cmcm* (No. 63).

Empirical formula	NdGa	NdGaH_0.9_	NdGaH_1.1_	NdGaH_1.2_	NdGaH_1.6_
CSD reference	2265528	2265531	2265532	2265530	2265529
Pearson symbol, *Z*	*oC*8, 4	*oC*8, 4	*oC*8, 4	*oC*24, 12	*oC*24, 12
*a* (Å)	4.4329 (12)	4.1855 (6)	4.1600 (3)	12.3380 (6)	12.332 (2)
*b* (Å)	11.246 (3)	11.914 (2)	12.0374 (9)	12.2710 (9)	12.264 (2)
*c* (Å)	4.1735 (11)	4.1857 (6)	4.1825 (3)	4.1691 (2)	4.1782 (7)
*V* (Å^3^)	208.1 (1)	208.7 (1)	209.44 (3)	631.2 (1)	631.9 (2)
Calculated density ρ (g cm^−3^)	6.83	6.81	6.79	6.75	6.75
Absorption coefficient μ (mm^−1^)	37.10	36.99	36.86	36.69	36.65
θ range for data collection (°)	3.623–31.719	3.420–31.609	3.385–32.069	2.341–32.472	2.342–39.415
*F*(000)	364	364	364	1092	1092
Range in *hkl*	−6 ≤ *h* ≤ 6	−5 ≤ *h* ≤ 6	−6 ≤ *h* ≤ 5	−18 ≤ *h* ≤ 18	−21 ≤ *h* ≤ 21
	−15 ≤ *k* ≤ 16	−17 ≤ *k* ≤ 16	−17 ≤ *k* ≤ 17	−18 ≤ *k* ≤ 14	−21 ≤ *k* ≤ 21
	−6 ≤ *l* ≤ 6	−6 ≤ *l* ≤ 6	−6 ≤ *l* ≤ 6	−6 ≤ *l* ≤ 5	−7 ≤ *l* ≤ 7
Total No. of reflections	2217	1820	1779	7143	7329
No. of independent reflections	220 (*R*_eq_ = 0.0296)	221 (*R*_eq_ = 0.0147)	227 (*R*_eq_ = 0.0289)	635 (*R*_eq_ = 0.0572)	1070 (*R*_eq_ = 0.0306)
No. of reflections with *I* > 2σ(*I*)	187 (*R*_sigma_ = 0.0180)	220 (*R*_sigma_ = 0.0091)	214 (*R*_sigma_ = 0.0182)	511 (*R*_sigma_ = 0.0325)	919 (*R*_sigma_ = 0.0217)
Data/parameters	220/10	221/9	227/9	635/21	1070/21
Goodness of fit on *F* ^2^	1.107	1.293	1.251	1.063	1.080
Final *R* indices [*I* > 2σ(*I*)]	*R*_1_ = 0.0147	*R*_1_ = 0.0105	*R*_1_ = 0.0156	*R*_1_ = 0.0276	*R*_1_ = 0.0295
	*wR*_2_ = 0.0193	*wR*_2_ = 0.0256	*wR*_2_ = 0.0308	*wR*_2_ = 0.0510	*wR*_2_ = 0.0651
*R* indices (all data)	*R*_1_ = 0.0225	*R*_1_ = 0.0107	*R*_1_ = 0.0179	*R*_1_ = 0.0470	*R*_1_ = 0.0376
	*wR*_2_ = 0.0193	*wR*_2_ = 0.0257	*wR*_2_ = 0.0317	*wR*_2_ = 0.0567	*wR*_2_ = 0.0683

**Table 2:** Atomic coordinates and equivalent isotropic displacement parameters for NdGaH*_x_* (*x* = 0–1.6) from SCXRD at 296 K

Atom	Site	*x*	*y*	*z*	*U*_eq_ × 100 (Å^2^)
NdGa					
Nd	4*c*	0	0.35806 (3)	1/4	1.02 (1)
Ga	4*c*	0	0.07009 (6)	1/4	1.13 (1)

NdGaH_0.9_					
Nd	4*c*	0	0.34572 (2)	1/4	0.77 (1)
Ga	4*c*	0	0.05794 (4)	1/4	1.14 (1)

NdGaH_1.1_					
Nd	4*c*	0	0.34524 (3)	1/4	0.71 (1)
Ga	4*c*	0	0.05698 (7)	1/4	1.00 (1)

NdGaH_1.2_					
Nd1	4*c*	0	0.33415 (5)	1/4	0.71 (2)
Nd2	8*g*	0.32999 (3)	0.35426 (3)	1/4	0.73 (1)
Ga1	4*c*	0	0.05939 (10)	1/4	0.83 (3)
Ga2	8*g*	0.34928 (7)	0.05844 (7)	1/4	0.96 (2)

NdGaH_1.6_					
Nd1	4*c*	0	0.33632 (3)	1/4	0.63 (1)
Nd2	8*g*	0.33074 (2)	0.35288 (2)	1/4	0.60 (1)
Ga1	4*c*	0	0.05919 (7)	1/4	0.78 (2)
Ga2	8*g*	0.34675 (6)	0.05837 (6)	1/4	0.99 (1)

**Table 3:** Crystallographic data and structure refinement for NdGaD_0.9_ and NdGaD_1.6_ (both space group *Cmcm*, No. 63), fractional atomic coordinates, site occupancy factor (SOF) and atomic displacement parameters at 20 K Values refer to data from WISH detector bank 3 at 2θ = 90°.

Atom	Site	*x*	*y*	*z*	SOF	*U*_eq_ × 100 (Å^2^)
NdGaD_0.9_, *a* = 4.1736 (9) Å, *b* = 11.916 (3) Å, *c* = 4.1816 (9) Å, *V* = 208.0 (7) Å^3^ and *R*_Bragg_ = 5.38						
Nd	4*c*	0	0.3449 (2)	1/4	1	0.45 (1)
Ga	4*c*	0	0.0568 (1)	1/4	1	0.65 (1)
D	4*c*	0	0.7485 (1)	1/4	0.91 (1)	1.7 (4)
						
NdGaD_1.6_, *a* = 12.297 (3) Å, *b* = 12.221 (3) Å, *c* = 4.1619 (8) Å, *V* = 625.4 (5) Å^3^ and *R*_Bragg_ = 8.48						
Nd1	4*c*	0	0.3550 (9)	1/4	1	1.28 (4)
Nd2	8*g*	0.33381 (9)	0.35211 (6)	1/4	1	0.41 (2)
Ga1	4*c*	0	0.0560 (1)	1/4	1	1.08 (4)
Ga2	8*g*	0.34728 (6)	0.06052 (8)	1/4	1	1.28 (3)
D1	4*c*	0	0.7498 (1)	1/4	1	3.5 (6)
D2	8*g*	0.1682 (1)	0.24893 (9)	1/4	1	3.0 (3)
D3	8*g*	0.18670 (9)	0.05154 (8)	1/4	0.890 (2)	3.5 (3)

**Table 4:** Relevant interatomic distances in NdGaD_0.9_ and NdGa_1.6_, *T* = 20 K, with estimated standard deviations in parentheses

NdGaD_0.9_				NdGaD_1.6_			
Atom pair			*d*(Å)	Atom pair			*d*(Å)
D1	Nd1	×2	2.368 (1)	D1	Nd1	×2	2.327 (1)
	Nd1	×2	2.382 (1)		Nd2	×2	2.396 (1)
Ga1	Ga1	×2	2.491 (1)	D2	Nd1		2.323 (1)
					Nd2		2.395 (2)
					Nd2	×2	2.420 (1)
				D3	Ga2		1.978 (1)
					Ga1		2.296 (1)
					Nd2	×2	2.404 (1)
					Nd2		2.450 (1)
				Ga1	D3	×2	2.296 (1)
					Ga1	×2	2.490 (1)
				Ga2	D3		1.978 (1)
					Ga2	×2	2.553 (1)
