# Supplementary material for: Revisiting the hydrogenation behavior of NdGa and its hydride phases
Source: J Appl Crystallogr. 2024 Feb 16;57(Pt 2):248–57. doi: 10.1107/S1600576724000554 (PMC11001401; doi:10.1107/S1600576724000554)
Supplement: Supplementary file 7 [file j-57-00248-sup7.pdf]

JOURNAL OF APPLIED CRYSTALLOGRAPHY

Volume 57 (2024)

Supporting information for article:

Revisiting the hydrogenation behavior of NdGa and its hydride phases

Vitalii Shtender, Johan Cedervall, Gustav Ek, Claudia Zlotea, Mikael Andersson, Pascal Manuel, Martin Sahlberg and Ulrich Häussermann

Revisiting the hydrogenation behavior of NdGa and its hydride phases

Vitalii Shtender,[1,*] Johan Cedervall,[1] Gustav Ek,[1] Claudia Zlotea,[2] Mikael S. Andersson,[1] Pascal Manuel,[3] Martin Sahlberg,[1] Ulrich Häussermann[4]

[1]Department of Chemistry – Ångström Laboratory, Uppsala University, Box 538, Uppsala 751 21, Sweden

[2]Univ Paris Est Creteil, CNRS, ICMPE, UMR 7182, 2 rue Henri Dunant, 94320 Thiais, France

[3]ISIS Neutron and Muon Facility, Science and Technology Facilities Council, Rutherford Appleton Laboratory, Oxford OX11 0QX, UK

[4]Department of Materials and Environmental Chemistry, Stockholm University, 106 91 Stockholm, Sweden

*Corresponding author: vitalii.shtender@angstrom.uu.se

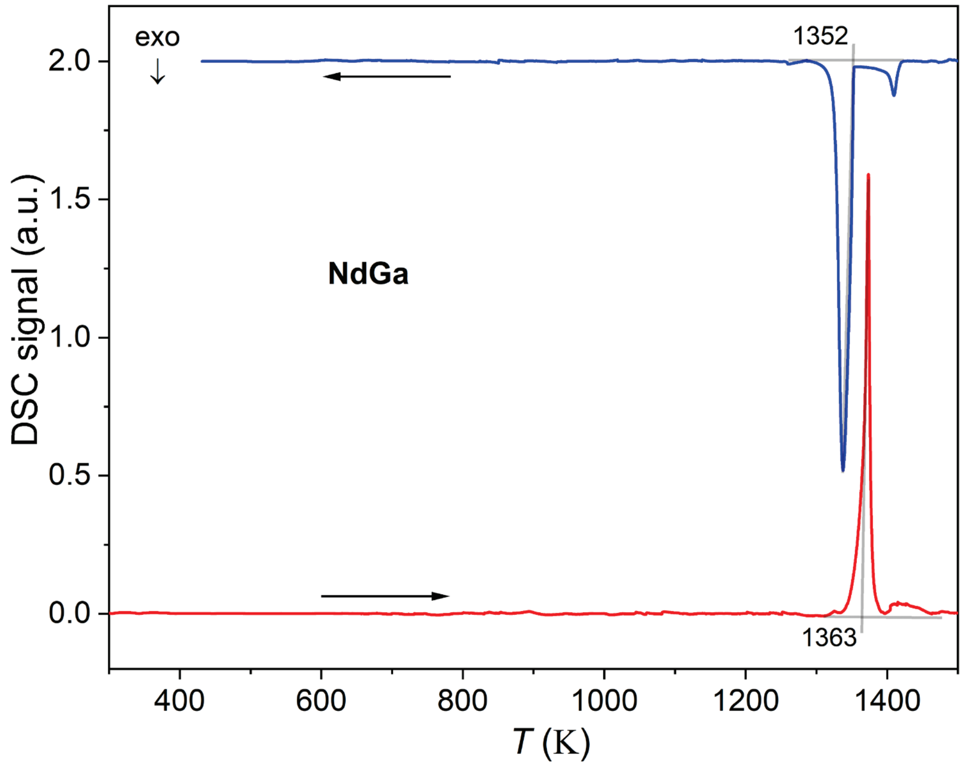

Figure S1. DSC spectrums for NdGa compounds with outlined onset temperatures.

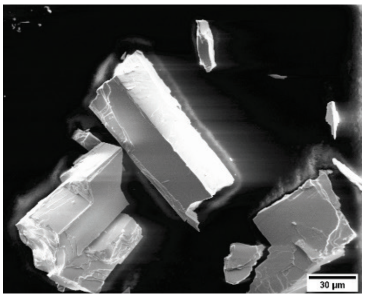

NdGa

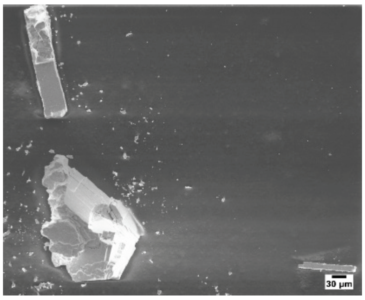

$NdGaH_{0.9}$

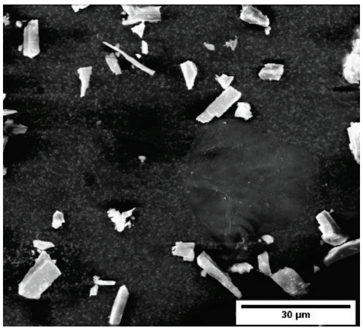

$NdGaH_{1.2}$

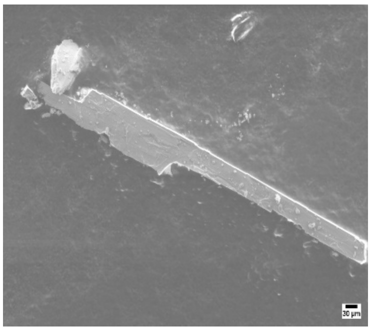

$NdGaH_{1.6}$

Figure S2. SEM images for $NdGaH_x$ (x = 0, 0.9, 1.2 and 1.6).

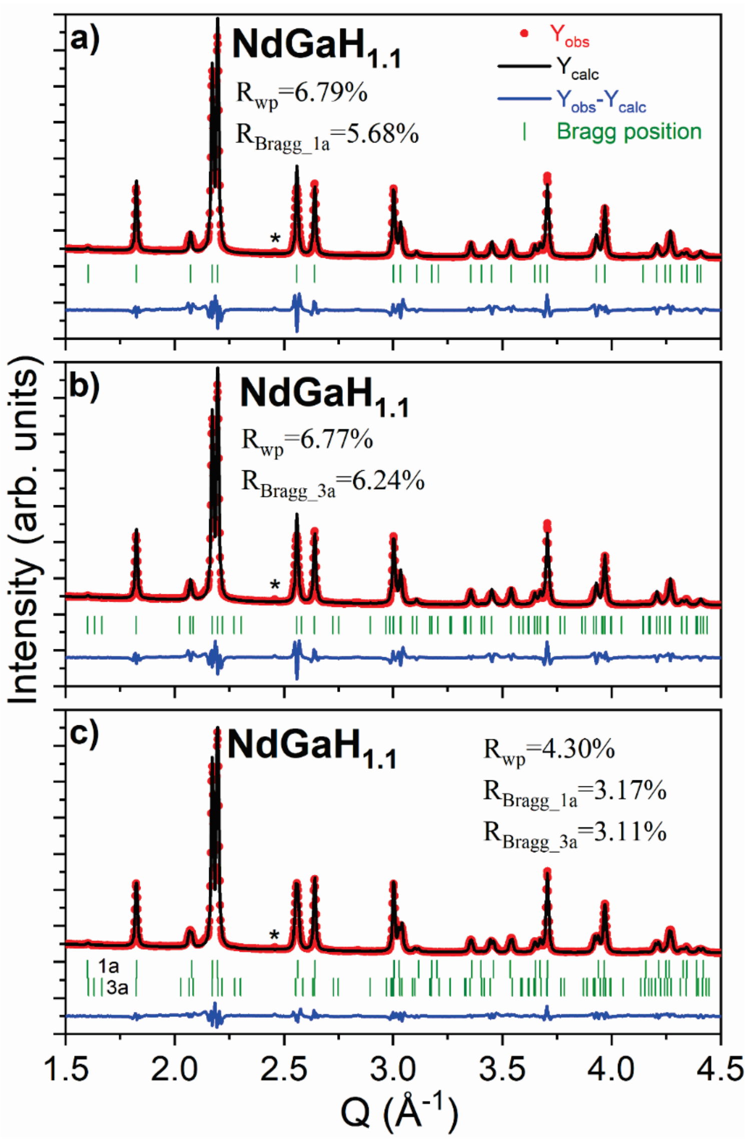

Figure S3. Rietveld refinement of $NdGaH_{1.1}$ sample. a) fitting the 1*a* CrB type structure b) fitting 3*a*-superstructure, c) fitting as two-phase mixture. * indicates the impurity of $TaGaO_4$.

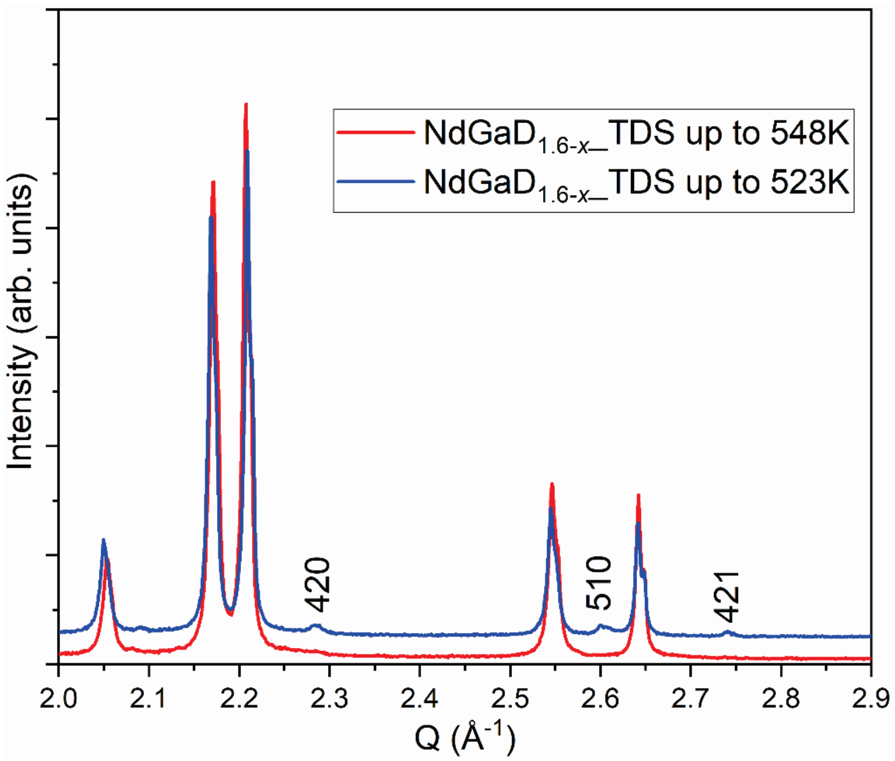

Figure S4. Comparison of two PXRD patterns of deuterides heated to 523 and 548 K. hkl's of supperlattice peaks are presented.

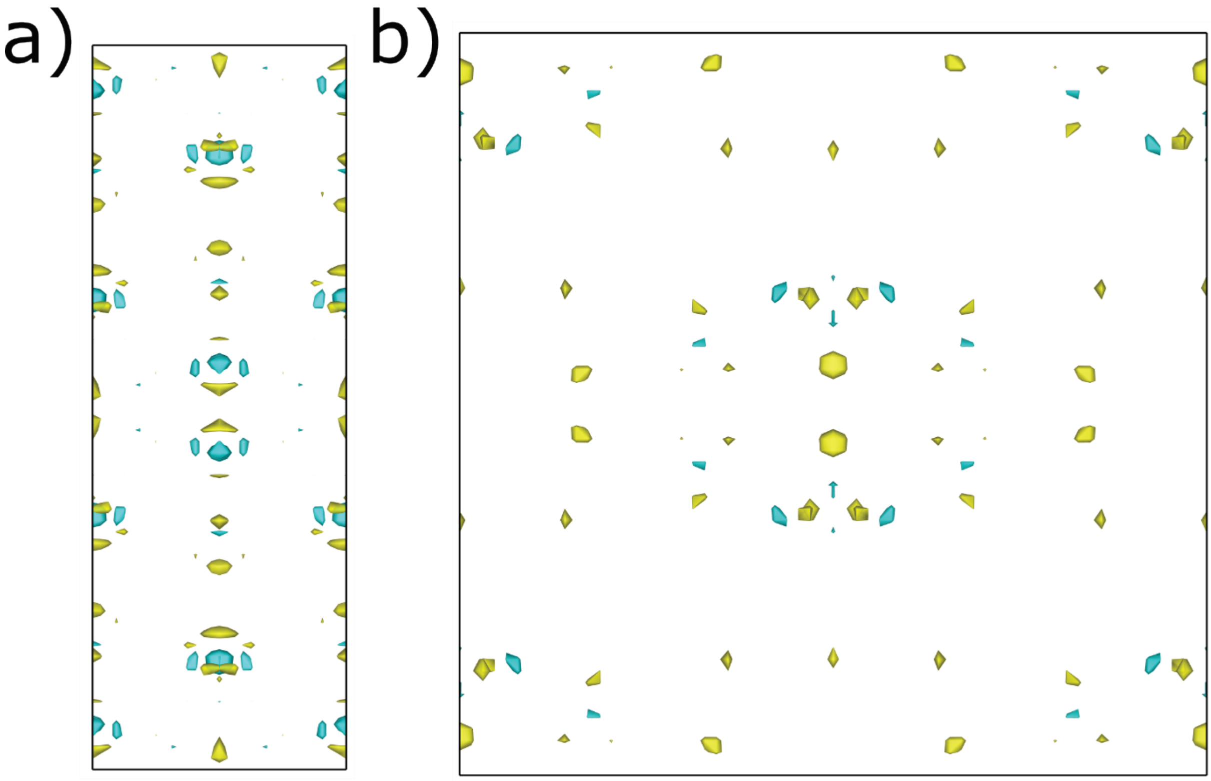

Figure S5. Fourier difference maps for a) $NdGaD_{0.9}$ and b) $NdGaD_{1.6}$. Isosurface levels are set to 0.1 fm/Å^3.
